# Organisational reporting and learning systems: Innovating inside and outside of the box

**DOI:** 10.1177/1356262215574203

**Published:** 2015-01

**Authors:** Mark Sujan, Dominic Furniss

**Affiliations:** 1 Warwick Medical School, University of Warwick, Coventry, UK; 2 UCL Interaction Centre, UCL, London, UK

**Keywords:** Patient safety, incident reporting, organisational learning, risk management, resilience

## Abstract

Reporting and learning systems are key organisational tools for the management and prevention of clinical risk. However, current approaches, such as incident reporting, are struggling to meet expectations of turning health systems like the UK National Health Service (NHS) into learning organisations. This article aims to open up debate on the potential for novel reporting and learning systems in healthcare, by reflecting on experiences from two recent projects: Proactive Risk Monitoring in Healthcare (PRIMO) and Errordiary in Healthcare. These two approaches demonstrate how paying attention to ordinary, everyday clinical work can derive useful learning and active discussion about clinical risk. We argue that innovations in reporting and learning systems might come from both inside and outside of the box. ‘Inside’ being along traditional paths of controlled organisational innovation. ‘Outside’ in the sense that inspiration comes outside of the healthcare domain, or more extremely, outside official channels through external websites and social media (e.g. patient forums, public review sites, whistleblower blogs and Twitter streams). Reporting routes that bypass official channels could empower staff and patient activism, and turn out to be a driver to challenge organisational processes, assumptions and priorities where the organisation is failing and has become unresponsive.

## Introduction

Policy makers widely acknowledge that modern healthcare systems may inflict preventable harm on patients.^1,2^ It is commonly suggested that around 1 in 10 patients admitted to hospitals around the world will suffer an adverse event, and that as many as half of these may be preventable.^3^ This causes needless harm to patients, and it represents an unnecessary additional financial burden on healthcare systems. In the UK, it has been estimated that preventable adverse events could cost the NHS £1bn annually in additional bed days, alone.^4^ The costs to the NHS associated with adverse drug events are estimated to be around £0.5bn–£1.9bn annually.^5^

In the UK, the National Reporting and Learning System (NRLS) was set up following the publication of the influential report ‘An Organisation With A Memory’ by the Department of Health.^1^ NRLS is a national incident reporting system that aims to systematically capture data about incidents in the NHS, and to provide an indication of the extent and the nature of harm that patients suffer in the NHS. The report also highlighted that the NHS needed to progress from a culture of blame towards an open, fair and just culture. Fear of punishment following errors represents a fundamental barrier to reporting, thus undermining this essential mechanism to enhancing patient safety. For example, the investigation into the Bristol Royal Infirmary deaths identified a deficient safety culture as a causal factor.^6^ More recently, the Francis report of the Mid Staffordshire Public Inquiry provided similar findings about a culture that was contributing to poor standards of care.^7^

Over the past 10 years, the NHS has adopted incident reporting as one of the key instruments for organisational learning.^8–10^ In addition to NRLS, which operates at a national level, organisations can also operate their own local incident reporting systems. The thinking behind incident reporting is that the analysis of reported incidents can offer valuable lessons about vulnerabilities in healthcare systems and about deficient organisational processes, which can reveal latent conditions for incidents.^11,12^ Such contributory factors can then be addressed before they combine to cause harm to patients.

The NRLS has had some impact in form of Rapid Response Reports, Patient Safety Alerts and Safer Practice Notices that raise awareness of system vulnerabilities based on analyses of incident report data. It was hoped that the role of the NRLS in improving patient safety would be as significant as that of the Aviation Safety Reporting System (ASRS) has been in the aviation sector. However, it has been suggested that NRLS has not achieved these aspirations.^13^ Research has provided insights into the barriers to successful learning from incident reporting, and the limitations of such an approach to organisational learning in the NHS. Barriers include lack of training in the use of incident reporting, usability problems of the systems that are used for reporting, uncertainty about what constitutes a reportable incident, blame culture and fear of consequences, lack of feedback and the absence of learning relevant to local practices.^14–17^ A major weakness of many incident reporting systems in the NHS is that they produce little actual change.^18^ The perceived lack of learning and absence of relevance to the local work environment may have a detrimental impact on the willingness of staff to contribute to incident reporting.^19^ This suggests that further research is required to develop approaches to organisational learning, which can complement incident reporting, and which are able to engage staff and generate actionable learning.^20^

## Learning from the ordinary

Organisational learning within the NHS relies largely on information derived from the analysis of serious untoward incidents and other harm events. In this respect, organisations are directing the focus of their learning on extraordinary (i.e. low frequency) events that cause patient harm. It is undoubtedly very important to understand what went wrong following any event where patients were harmed, but questions can be asked as to whether this is the only, or indeed, the best route into gathering information for improving the quality and safety of services. The problem with learning from such extraordinary events is that they frequently carry negative connotations of human error and blame. As a result, reporting is often done ‘to cover oneself’ rather than to contribute to organisational learning, and the events that get reported are typically restricted to well-known occurrences, such as patient falls. This limits the amount of useful information that can be generated by these approaches.

Within the Health Foundation project PRIMO (Proactive Risk Monitoring in Healthcare), an alternative (and complementary) approach to organisational learning was developed that aims to elicit a rich contextual picture of the local work environment, to move away from negative and threatening notions of errors and mistakes, and to encourage active participation and ownership with clear feedback for local work practices.^21,22^ The distinguishing feature of the PRIMO approach is that it focuses on learning from the ordinary, in this case the various hassles that practitioners experience in their everyday clinical work.

PRIMO is intended to be operated by local teams, e.g. the hospital pharmacy or individual wards. Staff are asked to contribute free-text narratives about something that caused them hassle during their working week. These staff narratives are anonymised and de-identified, and are entered into an electronic platform. The local PRIMO champion will do a preliminary analysis of each narrative, extracting both underlying causal factors, such as inadequate equipment, as well as the symptoms of these factors in the work environment, i.e. the actual hassle that people experience, such as having to spend excessive amounts of time chasing missing equipment. In this way, information is gathered from frontline staff about contributory factors that might at some point lead to patient harm if left unaddressed.

The benefit of learning from the ordinary hassle of everyday clinical work is that staff are usually very happy to report and to discuss these, because there is no fear of blame involved, and because staff would like to see their work environment improved. A further benefit is that some of the ordinary hassle can be addressed with limited resources and within a reasonable amount of time. This lends itself to create ownership among groups of staff, who can take responsibility for addressing and improving some of the hassle that has been identified. For example, in a hospital, dispensary staff reported that there was little space for pharmacists to do the final checking of medications. The local team then took this on, and the work environment was reorganised to create a dedicated space for this purpose.^20,21^ While it is important to address the underlying factors that might contribute to adverse events, this usually takes longer and is slow to show improvements at the local level. Focusing also on addressing the symptoms (i.e. the hassle) can contribute to engaging staff in the continuous improvement of safety and quality.

One of the hall-marks of the so-called High Reliability Organisations is that they keep the discussion about risk and safety going even in the absence of adverse events.^23^ Within PRIMO, the electronic platform offers the opportunity for staff to review all of the narratives, and to vote on both the extent of hassle they experience from symptoms as well as on the presence of the underlying factors using a social-media style ‘like’ approach. In this way, PRIMO might contribute towards building a community-based discussion of patient safety issues in the local work environment.

The evaluation of PRIMO in a radiology department and a surgical emergency assessment unit suggests that PRIMO can generate actionable learning that feeds into visible improvements in the work environment.^22^ Experience from implementing the PRIMO approach over a 12-month period in the two diverse settings further suggests that there are a number of common prerequisites that greatly influence the extent to which the PRIMO approach, as well as any other organisational learning tool, can contribute to successful proactive organisational learning and improvement. The identified and inter-related prerequisites are engagement by frontline staff, the composition of the improvement team, which needs to be sufficiently broad encompassing different staff roles, the buy-in and support from senior management, and the readiness of the organisation for quality and safety improvement.

## Learning by disrupting notions of the extraordinary

‘Serious untoward incidents’ and ‘never events’ have been adopted as labels to describe the most serious events in the NHS, which often result in patient harm and which should not have happened. This language emphasises the gravity and rarity of the event. This same language arguably provides some reassurance to staff and patients that these events are extraordinary, and unlikely to happen here, to me.

An alternative label from the safety literature, which provides a controversial substitute for a ‘never event’, is the ‘normal accident’.^24^ Perrow coined this term to describe how the myriad of small everyday disturbances, which are considered normal on their own, can coalesce to produce an accident. His thesis proposes that in complex systems these small disturbances are unavoidable, and eventually they will coalesce to lead to an accident. ‘Normal accident’ captures how nothing out of the ordinary caused the accident along the way, and it is only the normal disturbances coalescing which leads to the more remarkable outcome.

In a similar way, Errordiary in Healthcare also attempts to challenge notions of the extraordinary error, and provide an opportunity to debate normal risks with clinicians. Errordiary in Healthcare is a UCL Public Engagement Project, which invited clinical professionals, patients and the public to submit examples of error to an open public website. These contributions can be submitted directly through the website or by including the hashtag ‘#errordiary’ through Twitter. Over 130 volunteers have contributed to the Errordiary project so far with over 2,700 examples of error.^25^ These errors can be funny, frustrating or fatal. Workshops based on this work provide opportunity for learning through juxtaposing serious and less serious cases, e.g. mixing up similarly looking hair spray and body spray cans compared to mixing up similarly looking dye and glue bottles in surgery.^26^ Even though their outcomes are vastly different (hairspray on your body compared to a patient fatality) similar psychological causes can be attributed to these errors.^27^ Discussion about funny and everyday error can raise debate about more serious types in a non-threatening way, and increase mindfulness that these errors could happen here, to me, and to you.

## Learning from ‘going right’ rather than from ‘going wrong’

**Figure 1.**
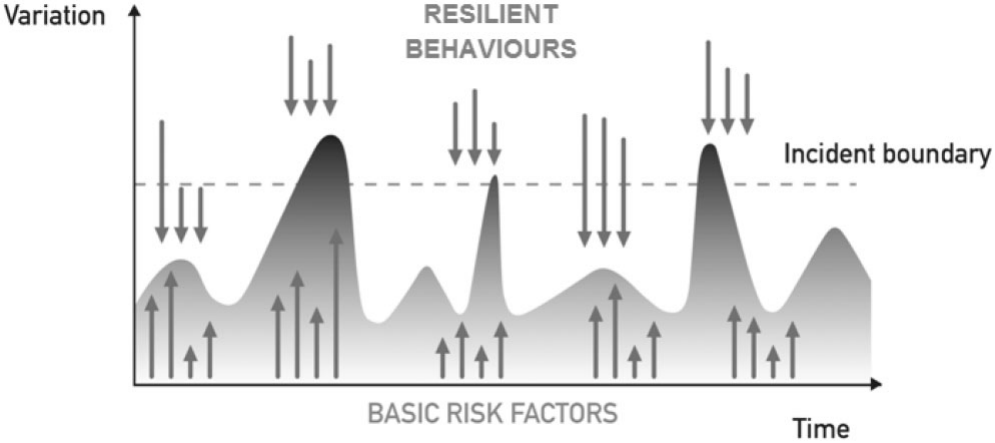
System dynamics model – the system is maintained under control by counterbalancing disturbances (upward arrows) with resilient behaviours (downward arrows).

So far, the work we have discussed on reporting and learning systems has revolved around different notions of incidents, hassles and disturbances. This reflects a preoccupation of putting right what appears to be failing or going wrong. However, taking inspiration from Resilience Engineering^28^ and the emerging notion of Safety-II^29^ (defined as the ability to succeed), we can also enhance safety management by fostering what a system does right and how it maintains performance despite vulnerability and adversity. The system dynamics can be visualised as shown in Figure 1, where resilient forms of behaviour support maintaining control of care delivery in the face of disruptive forces, such as lack of equipment or inadequate staffing levels.^17^

Both, PRIMO and Errordiary lend themselves to providing insights about how healthcare professionals deliver safe care in the face of disturbances and disruptions. Within PRIMO, the narratives that staff submit often contain not only descriptions of hassle, but also descriptions of how staff coped with the hassle. Such coping mechanisms or resilient forms of behaviour often include the sharing of information and personal negotiation to create a shared awareness, prioritisation of activities, and offering and seeking help.^30^ Errordiary also collects examples of resilience strategies, i.e. strategies used to minimise the likelihood or consequences of error.^31^ For example, strategies for taking medication include setting alarms, differentiating similar looking pills and having spare medication at work as well as at home.^32^ Workshops on this work also provide opportunity for learning through comparing similar cases, e.g. using coloured key rings to differentiate keys is similar to labelling intravenous infusion lines, so lines that lead to a patient can be easily identified.^26^ There is a possibility that sharing local and community relevant resilience strategies could help reduce clinical risk. Errordiary can be considered an embryonic architecture, in structure and concepts, for such a system. With incident reporting such learning typically is not available. This is because incidents represent situations that have broken down, i.e. situations where the coping strategies have proved insufficient to maintain the system (or care delivery process) under control. However, looking at ordinary, everyday clinical work allows a better understanding of the system dynamics at play.

## Innovations outside of the box

Innovations in reporting and learning systems can come from different threats and opportunities inside and outside of normal organisational structures. Policy and the need for organisational learning can drive the development and implementation of internal reporting and learning systems, as was the case with the NRLS. However, looking ahead we see a greater role for reporting systems outside of the organisational structure, which might bypass official channels and restrictions. Trends in other areas allude to developments, mostly facilitated by the affordances of modern technology. For example, patient and staff activism can be more easily operationalised through social media and blogs. Similar to how a schoolgirl captured public attention by blogging about the poor standard of her school dinners,^1^ patients and staff could campaign against poor nutritional standards or other failing standards of institutions. External quality reporting and rating systems are rife in the product and service industry, like TripAdvisor^2^; further research could investigate whether these informal and external rating systems could be a model for the health service. Similar systems that rate individuals, such as ratemyprofessor,^3^ might be applied to GPs, dentists and other healthcare professionals. Also, systems are now in place to give a voice to disgruntled staff, an extreme example of which is WikiLeaks.^4^ Such systems might facilitate staff raising concerns about patient care where they feel unable to do so through organisational channels, which could have been useful in relation to the Mid Staffordshire case.

A further area where additional research is required is in understanding the factors that enable or inhibit the successful implementation of such approaches to organisational learning. Approaches to evaluation rooted in realism emphasise the need to understand the mechanisms and the context of change.^33^ Stevens emphasises the importance of context in improvement reports highlighting the need for reflection of the interaction between improvement strategy and the unique context.^34^ Further research should, therefore, aim to identify and describe the factors that contribute to successful organisational learning across a range of different settings.

## Conclusion

Healthcare providers should seek out alternative approaches to complement their established organisational learning processes. Examples of approaches that focus on everyday clinical work have already been developed and tested. Other examples can be found outside of the health sector, and this could provide inspiration for healthcare-specific learning processes. How organisations respond to these external reporting mechanisms, whether they perceive them as a threat or opportunity, and how they embrace learning from this remain to be seen, but there is an opportunity for both policy makers and researchers to look to these developments and formulate and test potential lessons for healthcare.

## Notes

1. http://www.bbc.co.uk/news/uk-scotland-glasgow-west-18454800

2. http://www.tripadvisor.co.uk

3. http://www.ratemyprofessors.com

4. https://wikileaks.org
